# Impact of Environmental Regulations on Export Trade—Empirical Analysis Based on Zhejiang Province

**DOI:** 10.3390/ijerph191912569

**Published:** 2022-10-01

**Authors:** Juan Huang, Ziyi Wu

**Affiliations:** 1 School of Economics, Hangzhou Dianzi University, Hangzhou 310018, China; 2 School of Slavonic and East European Studies, University College London, London WC1E 6BT, UK

**Keywords:** environmental regulations, export trade, extended gravity model, green economy

## Abstract

There is a close connection between export trade and environmental regulations. How to realize the green development of export trade under the constraint of environmental regulation policy is a controversial topic in both theoretical research and practice. Considering the leading role of Zhejiang export trade in China, based on the extended gravity model, this paper attempts to explore the impact of environmental regulations on export trade using the panel data of Zhejiang Province together with that of 18 major “Belt and Road” trading countries (regions) from 2004 to 2016. It provides a theoretical basis for promoting the coordinated development of environmental protection and export trade. This not only has theoretical and practical significance for various regions in China but also for other countries and regions in the world when formulating environmental regulation standards and implementation intensity. The empirical results show that there is a U-shaped relationship between environmental regulations and the development of export trade; namely, the former suppresses the latter before promotion happens. Specifically, environmental regulation could increase the cost of export products and curb the development of export trade in the short term. On the other hand, it encourages enterprises to carry out technological innovation and improve efficiency and competitiveness, thus contributing to the development of export trade in the long term.

## 1. Introduction

Currently, the challenges of the ecological environment are becoming increasingly serious with the expanding volume and openness of the world economy; a number of countries are pursuing economic growth while attaching importance to the environment. The relationship between the environment and trade is close, with widespread attention from the international community, proven by more than 20 multilateral environmental agreements with a direct impact on trade. Additionally, environmental and trade-related issues have been put on the agenda of the WTO multilateral trading system. In May 2017, the Chinese government hosted the “Belt and Road” International Cooperation Summit Forum, in which President Xi proposed a green concept and regarded the “development of green trade as well as sustainable production and consumption” as one of the major tasks of green “belt and road” construction.

At present, China is undergoing critical economic and trade transformation, and eliminating worsening environmental pollution is crucial for the transformation’s success. The report of China’s 19th National Congress also proposed to accelerate the formation of an ecological civilization system. Therefore, it is urgent to solve environmental pollution while maintaining the stable growth of foreign trade. Zhejiang Province, as a leader and powerful province in China’s export trade as well as a leader in cross-border e-commerce trade, represents the future of China’s foreign trade development. To be more specific, the total trade volume of Zhejiang Province reached CNY 3.38 trillion, accounting for 10.5% of China’s total imports and exports in 2020, among which the total export reached CNY 252 trillion, accounting for 74.5% of its trade volume and 14% of China’s total export volume. Moreover, the export scale of Zhejiang Province has always been in the top three in China so far. While encouraging green trade, Zhejiang Province is actively involved in upgrading its industrial structure and changing its trade mode, constantly reinforcing its implementation of environmental regulations. As the main industrial emission, SO_2_ has shown a downward trend since 2004, and the decade between 2015 and 2016 witnessed its most significant decline from 52.4 to 24.5 tons and down to 50,000 tons in 2020. Meanwhile, the revenue of pollutant discharge in Zhejiang Province showed an overall growth trend between 2004 and 2020, a more rapid increase from 2004 to 2008, and fluctuations from 2009 to 2020. This shows that environmental regulation in Zhejiang Province has constantly improved and played a positive role in environmental protection. However, environmental regulation is a “double-edged sword”. Will energy savings and emission restrictions reduce the competitiveness of export trade? Can these stringent environmental regulation policies achieve a win–win situation for the environment and export trade? Does the “Porter Hypothesis Effect” exist in China? With above questions in mind, this paper takes Zhejiang Province—one of China’s leading export provinces with the strictest environmental regulations, as an example, and conducts an empirical study by selecting panel data between Zhejiang Province and 18 major trading countries (regions) along “belt and road” from 2004 to 2016, it aims to identify whether environmental regulation inhibits the development of export trade and test the impact of environmental regulation on export trade to provide an insight for the coordinated development of environmental protection and export trade. The research result is theoretically and practically significant for both China and other countries or regions worldwide when formulating environmental regulations and their implementation.

## 2. Literature Review

Currently, the academic research on the impact of environmental regulation on export trade continues to deepen. Generally speaking, there are three main points of view:

The first view is that environmental regulation has a positive impact on the development of export trade. In other words, environmental regulation promotes the development of export trade, and proper environmental regulation stimulates innovation and the innovation of enterprises in export countries, contributing to the increased comparative advantages and competitiveness of products in the international market. Porter and van der Linde (1995) argued that “properly designed” environmental regulations encourage enterprises in exporting countries to innovate, improve productivity and product quality, as well as offset production costs for environmental protection, enabling export products to be more competitive [1]. Frankel and Rose (2005) believed that, in view of development, if future products in the world tend to be environmentally friendly, then countries that first initiate environmentally friendly technological innovation will gain comparative advantages in the future global market [2]. Mary and David (2011) established a general equilibrium model based on the environmental regulation of coal-fired power plants to study the relationship between environmental regulation, technological progress, and international trade in an open economy. The results showed that in large countries, strict environmental regulation promotes invention and environmentally friendly technology, ultimately contributing to the development of a country’s import and export trade [3]. Wang (2010) found that strict environmental regulation can stimulate enterprise innovation and enhance trade competitiveness, thus promoting the growth of foreign trade and achieving a triple-win situation for the environment, trade, and sustainable development as a result [4]. Yu and Sun (2017) [5] and Shi et al. (2017) [6] uncovered the positive effect of environmental regulation on international competitiveness and urban economic growth by analyzing panel data of 28 manufacturing industries in China and using the differences-in-differences method, and this effect was found to gradually climb with time and the progress of environmental regulation implementation. Wang Yi et al. (2019) used the double-difference method to conduct an empirical analysis on the relationship between environmental regulation and the domestic value-added rate of exports, and the results showed that China’s environmental regulation could significantly improve the domestic value-added rate of enterprises’ exports [7]. Gao and Yuan (2020) found that environmental regulation significantly improved the technical complexity of enterprises’ export by using database of Chinese industrial enterprises [8]. Ouyang Qiang et al. (2021) constructed a quintile regression model to analyze the impact of environmental regulation on export trade by using panel data of 30 provinces in China, and the results verified the conclusion that the “Porter hypothesis” exists in China [9]. Cherniwchan and Taylor (2022) concluded that although pollution-haven effects brought by environmental regulation are well-documented, there are no credible estimates to understand if it is larger or smaller than other factors [10].

The second view argues that environmental regulation restrains the development of export trade, and strict environmental regulation internalizes an enterprise’s external costs and reduces its profits, thereby cutting the comparative advantages of products. Dean (2002) [11] and Mulatu et al. (2004) [12] both agreed that strict environmental regulation would lower the comparative advantages of their export products and ultimately result in the transfer of their export trade to other countries and produce an “environmental regulation cost”. Fu and Li (2010) [13] and Zhou et al. (2016) [14] also point out that environmental regulation has a significant negative impact on the location distribution of FDI, e.g., outward transfer of pollution-intensive industries. Based on the extended gravity model, Ren and Huang (2015) analyzed China’s export data with 37 trading partners and concluded that the intensity of environmental regulation has a significant negative correlation with export trade; the higher the environmental regulation intensity, the greater the negative impact on export trade [15]. Shi and Xu (2018) applied the triple-difference method to estimate the impact of environmental regulation on firm exports and found that in pollution-intensive industries, stricter environmental regulation reduced the possibility and volume of enterprises’ export [16]. Zhang (2019) referred to clean production standards implemented in China in 2003 based on a database of Chinese industrial enterprises and found that environmental-regulation policies have a negative impact on enterprises’ export in the short term [17]. He and Lu (2019) pointed out that the tightened environmental regulations may also add a burden to enterprises, hinder their investment in production and technology, and internalize their environmental costs, which are detrimental to enterprises’ export trade [18]. Cherniwchan and Najjar (2022) found that for the most affected manufacturers, regulation reduced export volumes by 32 percent and increased the likelihood that plants stop exporting by 5 percentage points [19]. 

The third view is that environmental regulation has no obvious influence on the development of export trade. Peter Neary (2006) [20] and Dean and Lovely (2008) [21] argue that environmental regulation has no obvious impact on the transfer of pollution-intensive industries because import and export trade are affected by various factors; one single factor cannot affect the trend of trade. Arouri et al. (2012) used a gravity model to test whether strict environmental regulation implemented by Romania has an impact on the competitiveness of export products, and the results showed that there was no obvious connection between them [22]. Similarly, Dechezlepretre and Glachant (2014) used panel data of OECD countries from 1994 to 2005 and studied the impact of energy policies both at home and abroad on wind power innovation and trade and reached the same conclusions [23].

Based on empirical analysis, Tang (2014) [24], Du and Li (2016) [25], and Wei and Zhong (2016) concluded that there is a U-shaped relationship between environmental regulation and comparative advantage in various industries of China [26]. That is, before an optimum point appears, the comparative advantages of environmental regulation and China’s industries are negatively related, while the correlation becomes positive after the point appears. Li and Ramarkrishnan (2018) explored the relationship between three different types of environmental regulations (command and control regulation, market regulation, and informal regulation) and environmental performance, and the results showed that the relationship between them was not significant either linearly or nonlinearly [27].

Based on the previous research, impact studies of environmental regulation on export at home and abroad focus on empirical analysis from the perspective of various country or industry, but an analysis of data adopted from certain regions are rare, and regional differences, therefore, are ignored. However, there are great regional differences in China; for example, the economy and trade are more developed, and environmental regulation has advanced fast in coastal areas in East China. This paper selects Zhejiang Province, a typical representative of eastern coast economies, as a sample for empirical analysis, aiming to explore the effect of regional environmental regulations on export while shedding light and providing a policy reference for regional economic development.

## 3. Effect Mechanism

The effect mechanism of environmental regulation on export trade refers to the process in which the formulation and implementation of environmental regulations affect the development of factors such as trade cost, innovation, and structure. In this part, the effect of environmental regulation on export trade will be analyzed from three aspects: trade cost, trade innovation, and trade structure.

### 3.1. Effect Mechanism of Environmental Regulation on Trade Cost

The effect of environmental regulation on trade costs means that due to the ongoing conflict between the environment and trade, the government must implement environmental regulations to coordinate the relationship between them, and the environment is introduced into the production process, resulting in increased production cost and export cost for enterprises and reduced market competitiveness. The increased trade costs occur in three stages: the pre-production stage, the in-production stage, and the post-production stage.

#### 3.1.1. Pre-Production Stage

The effect of environmental regulation on trade costs is reflected in regular investment in fixed assets and the purchase of raw materials. To satisfy environmental protection requirements set by the government, enterprises have to upgrade their machine and equipment and introduce new and clean technology; meanwhile, environmental protection leads to a rise in raw material prices, especially for materials needed for resource-intensive products, which leads to increased production costs.

#### 3.1.2. In-Production

The effect of environmental regulation on trade costs is reflected in the fact that polluters need to pay for environmental-pollution behaviors, and enterprises need to purchase pollution-discharge permits, pay environmental taxes, pollution-discharge taxes, fines, environmental permit amortization fees, etc. In addition, environmental regulations make the production process more complex, which imposes higher requirements on production workers and management methods, and ultimately leads to an increase in labor costs and management costs. Therefore, the internalization of environmental regulation is mainly reflected in this stage.

#### 3.1.3. Post-Production Stage

The effect of environmental regulation on trade costs is mainly reflected in product-circulation costs. In order to meet the requirements of protecting the environment, enterprises need to design environmentally friendly product packaging and recycle waste.

### 3.2. Effect Mechanism of Environmental Regulation on Trade Innovation

Reasonable environmental regulation will effectively urge enterprises to carry out a series of innovative activities. By using the innovation compensation effect and first-mover advantage, enterprises can improve their competitiveness and realize the coordinated development of the environment and trade, which is supported by the well-known “Porter hypothesis”. The innovation compensation effect refers to improving the production efficiency of enterprises through innovation, thereby partially or even fully offsetting the costs caused by environmental regulation. The first-mover advantage refers to the first technological innovation, which gives the enterprise a leading position in environmental protection technology and ultimately makes the enterprise products gain comparative advantages and promote exports. The impact of environmental regulation on trade innovation includes:

#### 3.2.1. Compensating for the Costs of Environmental Internalization

The cost of environmental regulation is the “economic cost” of protecting the environment. If the enterprise is profit-oriented, it will find ways to offset the costs caused by environmental regulations, thereby increasing profit margins and maximizing profits. One of the most effective ways of achieving this is to carry out technological innovation. In addition, due to the gradual strengthening of environmental regulations and the increase in the prices of some raw materials, enterprises will carry out research and development of new recyclable and clean raw materials to reduce costs and protect the environment.

#### 3.2.2. Market Demand and Market Access

Under the influence of the concept of sustainable development, people’s demand for green products has greatly increased. Therefore, if enterprises want to seize the market, they must meet the needs of consumers and innovate in product packaging and environmental protection performance and services. At the same time, the deteriorating environment has raised the threshold for market access. Countries have stricter environmental standards for imported products. Enterprises promote the development of overseas markets by enhancing green innovation.

#### 3.2.3. Business Competition

Environmental regulation has produced more intense competition among enterprises, and the fierce competition environment has effectively stimulated enterprises to carry out innovation activities. In order to win the market, enterprises must actively carry out green innovation to improve their innovation and increase research and development in terms of management, production technology, product packaging, etc.

### 3.3. Effect Mechanism of Environmental Regulation on Trade Structure

According to the analysis of economic theory, strict environmental regulation will have an impact on the behavior and performance of enterprises, thereby affecting the industrial structure. The impact of the development of environmental regulation on trade structure has gradually emerged, mainly through the optimization and upgrading of the industrial structure and regional transfer.

#### 3.3.1. Optimization and Upgrading of Industrial Structure

Environmental regulation increases the production cost of pollution-intensive industries. An environmentally sensitive industry’s competitiveness is significantly affected by the endowment of environmental factors. First, the increase in environmental costs reduces the comparative advantage of such commodities. Second, with the gradual expansion of the green market and stricter environmental standards, environmentally friendly products will win the favor of more consumers and a broader market. Therefore, profit-oriented enterprises are willing to increase investment in the production of green products to maximize their benefits. Furthermore, the implementation of environmental regulations not only contributes to the energy conservation and emission reduction of pollution-intensive industries but also effectively promotes environmentally friendly industries while suppressing the development of pollution-intensive industries. In addition, the government and enterprises will introduce advanced environmental protection technologies and equipment and increase research and development and investment in clean energy and new green technologies to achieve optimal configuration within the industry. In short, with the intensification of environmental regulation, pollution-intensive industries will be gradually replaced by green and clean industries.

#### 3.3.2. Regional Transfer

According to the Pollution Paradise Hypothesis, environmental regulation will make pollution-intensive industries move from countries with stricter environmental regulations to countries with looser environmental regulations, thereby reducing costs and increasing profit margins. Due to the various levels of economic development in different countries, developed countries pay more attention to sustainable development and implement stricter and better environmental regulations, while developing countries are willing to lower environmental standards and produce polluting products to gain international competitiveness, so the implementation of environmental regulations is especially important. This may lead to the transfer of pollution-intensive industries from developed to less-developed regions. Tanaka et al. (2022) provide a salient example of a pollution-haven effect involving a developed and a developing country [28].

### 3.4. Research Hypothesis

In view of the above analysis, it can be concluded that environmental regulation will both promote and inhibit the development of export trade, so it cannot simply be said that environmental regulation will have a positive or negative impact on it, but should be based on the positive effect, and the contrast between the negative effect forces should be determined. Therefore, in view of the above analysis, combined with the previous research experience, the following assumptions are made about the impact of environmental regulation on the export trade of Zhejiang Province, which also pave the way for the empirical section below:

**Hypothesis** **1.** *In the initial stage, the implementation of environmental regulations will lead to the transformation of environmental costs from external to internal costs, and enterprises cannot implement effective measures to compensate for the additional production costs in the short term, which will lead to increased production costs and decreased competitiveness. This will have a negative impact on export trade; that is, the negative effect will be greater than the positive effect.*

**Hypothesis** **2.** *After a period of development, in order to maximize its benefits, enterprises will carry out technological innovation and structural upgrades to offset the negative impact of environmental regulations on trade costs. The advantage is enhanced. Therefore, during this period, environmental regulation will have a positive impact on export trade; that is, the positive effect will be greater than the negative effect.*

## 4. Empirical Model

Based on the existing research at home and abroad, this paper uses the extended gravitational model to analyze the impact of environmental regulation on export trade. This gravity model was proposed by Tinbergen in 1962 and introduced into the study of international trade. The basic idea of this model is that the level of bilateral trade is positively correlated with the gross production of both sides. Under various assumptions, the extended gravity model has been widely used by many scholars to explore the impact of various trade cost factors (such as distance, international trade policy, and national trade margin) on bilateral trade flows. It can be seen that the gravity model is a good econometric model to study the impact of environmental regulation on export trade. Therefore, this paper is based on the traditional gravity models of Anderson and van Wincoop (2003) [29] while adding environmental regulation as explanatory variables and related control variables to investigate the relationship between the trade in Zhejiang Province and its environmental regulation.

### 4.1. Model Establishment

According to Anderson and van Wincoop (2003) [29], the core equation of the gravity model is:

(1) $$y_{\mathrm{ij}}=\frac{x_{i}x_{j}}{x^{w}}{\left(\frac{T_{\mathrm{ij}}}{P_{i}P_{j}}\right)}^{1-\alpha }$$

where i and j represent two countries (or regions), y_ij_ indicates the import amount of country (or region) j from the country (or region) i, x_i_ and x_j_ denote the nominal income of consumers of i and j, x^w^ implies the nominal income of the world, and T_ij_ is the transportation cost of the “iceberg”. p_i_ and p_j_ are the consumer price indices of the invariable substitution elastic utility functions of countries (or regions) i and j, respectively. Because p_i_ and p_j_ cannot be observed in real life, the first-order logarithmic linear Taylor transformation of their function forms is necessary, and logarithms are taken on both sides to obtain the following:

(2) $${\mathrm{lny}}_{\mathrm{ij}}=a_{0}+{\mathrm{lnx}}_{i}+{\mathrm{lnx}}_{j}-\left(\sigma -1\right)T_{\mathrm{ij}}+\left(\sigma -1\right)\left(\Sigma_{k=1}^{N}\theta_{k}{\mathrm{lnT}}_{\mathrm{ik}}-\frac{1}{2}\Sigma_{k=1}^{N}\Sigma_{m=1}^{N}\theta_{k}\theta_{m}{\mathrm{lnT}}_{\mathrm{km}}\right)+\;\left(\sigma -1\right)\left(\Sigma_{k=1}^{N}\theta_{k}{\mathrm{lnT}}_{\mathrm{kj}}-\frac{1}{2}\Sigma_{k=1}^{N}\Sigma_{m=1}^{N}\theta_{k}\theta_{m}{\mathrm{lnT}}_{\mathrm{km}}\right)$$

where $a_{0}=-{\mathrm{lnx}}^{w},{\;\Sigma }_{k=1}^{N}{\;\Sigma }_{m=1}^{N}\theta_{k}\theta_{m}{\mathrm{lnT}}_{\mathrm{km}}$ remains unchanged and x_i_ and x_j_ are available. This equation can be used for empirical analysis only by knowing the determinants representing the unobservable external multilateral trade cost T_ij_. According to the traditional theory of international trade, geographical distance, population size, and land area are the factors affecting trade costs, and the explanatory variables in the traditional gravity model usually include these factors. However, some scholars maintain that the environmental regulation of exporting countries may affect their comparative advantages and thus have a negative impact on their export trade. This is one of the exogenous multilateral trade constraints in terms of trade cost. Therefore, this paper includes environmental regulation factors as explanatory variables affecting export trade in the extended gravity model. Furthermore, based on the existing research of Li and Li (2012) [30] and Liao and Xie (2017) [31], considering that the impact of environmental regulation on trade comparative advantage is non-linear, this paper introduces the second term of environmental regulation into the existing model. Zeng et al. (2020) point out FDI is the main factor in the rapid development of Zhejiang’s open economy [32], which also affects the export; this paper views the direct investment (FDI) of trading partners in Zhejiang Province, the human resources of Zhejiang Province, and capital level (RL) as the control variables. After mathematical calculation and model expansion, the unobservable trade cost T_ij_ model is transformed into a logarithmic linear equation of observable trade constraints. After simplifying and adjusting the formulas mentioned above, the final panel model of this paper is as follows.

(3) $${\mathrm{lnEX}}_{\mathrm{jt}}=a_{0}+a_{1}{\mathrm{lnX}}_{\mathrm{it}}+a_{2}{\mathrm{lnX}}_{\mathrm{jt}}+a_{3}{\mathrm{lnpop}}_{\mathrm{it}}+{\mathrm{lnpop}}_{\mathrm{jt}}+a_{5}{\mathrm{lndist}}_{t}+a_{6}{\mathrm{lnland}}_{\mathrm{jt}}+a_{7}{\mathrm{lnRL}}_{\mathrm{it}}+\;a_{8}{\mathrm{lnFDI}}_{\mathrm{it}}+a_{9}{\mathrm{lnER}}_{\mathrm{it}}+a_{10}{\left({\mathrm{lnER}}_{\mathrm{it}}\right)}^{2}+\varepsilon_{\mathrm{ij}}$$

where ln denotes logarithm, i indicates Zhejiang Province, t represents year, j is trading partner country (i.e., importing country), EX means export, X denotes GDP, pop indicates population, dist represents distance, land denotes land area, RL indicates human capital, FDI is the direct investment, ER is the degree of environmental regulation, and ε denotes the error term.

### 4.2. Data

This paper uses performance-based indicators, such as major pollutant emissions, to measure the level of environmental regulation (ER) in Zhejiang Province. The per capita industrial SO_2_ emissions indicate the differences in the intensity of environmental regulation. The level of human capital (RL) is measured by the proportion of scientific and technological personnel in the total number of employees. The distance variable is expressed by the distance between the capital of each country and the capital of Zhejiang Province. The other variables, such as GDP, FDI, distance, and land area, can be obtained directly. Based on the principle of scientific data and availability, all the variables involved in this paper are mainly derived and calculated from the Zhejiang Statistical Yearbook, the Zhejiang Foreign Trade Yearbook, Zhejiang Natural Resources, the Environment Statistical Yearbook, the World Bank World Development Index (WDI) database, and the Zhejiang Environmental Bulletin. The observation period is 2004–2016.

Since the existence of variable non-stationarity will affect the accuracy of regression coefficients, this paper first carries out a variable stationarity test. As can be seen in Table 1, each variable passes the LLC test and AD-Fisher test. Therefore, all variables are stationary. In addition, the statistical description of the variables is shown in Table 2.

### 4.3. Empirical Estimation

In order to control the endogeneity of variables, this paper utilizes the first-order lag term of export volume of Zhejiang Province (L.EX) as a tool variable on the basis of Formula (3), uses the GMM method to estimate panel data dynamically, and uses the AR (1) test, AR (2) test, and Sargan test to judge whether the tool variables of the model are valid or over-recognized. In addition, the stepwise regression method is used to estimate the distribution of the model to better overcome the influence of multiple collinearities. The regression test results are shown in Table 3.

The results of AR (1) and AR (2) in Table 2 are all at the 10% significance level, and the difference in the random error terms of all estimation models has first-order autocorrelation, but there is no second-order autocorrelation. The results of the Sargan test are all at the 10% significance level. Therefore, all estimation models conform to the original hypothesis that the over-recognition constraint is effective; that is, these variables are not related to the perturbation term. It can be concluded that the SYS-GMM estimation results in this paper are consistent and reliable. In the GMM estimation, the regression coefficient of the first-order lag term of the explained variable EX is significantly positive in all estimation models, indicating that the design of the dynamic panel model in this paper is reasonable. The specific analysis of each variable is outlined in the following.

#### 4.3.1. The Impact of Explained Variable

Environmental regulation: in the process of introducing other variables gradually, the coefficients of primary and secondary terms of lnER are significantly positive in all five models, and they are significant at the level of 1% or 5%. This shows that environmental regulation has a U-shaped dynamic effect on the export of Zhejiang Province; that is, there is an “inflexion point”. On the left side of the inflection point, the volume of export trade decreases with the increase in the intensity of environmental regulation; on the right side of this point, the volume of export trade increases with the increase in the intensity of environmental regulation. According to the estimated results of model (5), we calculate that the inflection point is about −3.86, where this U-shaped dynamic impact can occur. We believe that in the short term, environmental regulation reduces the comparative advantages of products and inhibits the development of export trade since the former internalizes environmental costs and increases costs. On the other hand, environmental regulation exerts a negative impact on the development of export trade in the long term, as it is conducive to stimulating enterprises to carry out technological innovation and improve their productivity through compensation effects, contributing to progress in their comparative advantages and the development of export trade. Since this paper uses performance-based indicators (i.e., major pollutant emissions) to measure the level of environmental regulation (ER) in Zhejiang Province, the lower the pollutant emissions, the higher the level of environmental regulation, so we can understand that the environmental regulation level calculated from the emission of pollutants will inhibit export trade until it is higher than −3.86, but as the intensity of environmental regulation exceeds −3.86, environmental regulation will promote the development of export trade. At present, the level of environmental regulation in Zhejiang Province is still before the inflection point.

#### 4.3.2. The Impact of Control Variables

GDP: both the GDP of Zhejiang Province and its trading partners have a positive impact on the export of Zhejiang Province. These two variables have a positive impact on the export of Zhejiang Province at a significant level of 1% or 5% in all models; this result fits the inference of the gravity model. That is, the higher the degree of bilateral GDP development, the closer bilateral trade contacts are. This is because a higher GDP means a larger market size, which promotes export.

Population: the coefficients of population size variables of Zhejiang trading partner countries are positive in all five models and show different degrees of significance. To be more specific, the population size of Zhejiang Province is positive in Models (1), (2), and (3) but negative in Models (4) and (5). From this, we can conclude that the impact of population size on export trade is uncertain. This is because a larger population size possibly means that the region has a higher level of human capital or a larger market capacity, which is more likely to promote the development of export trade. On the other hand, a larger population size tends to facilitate domestic trade and thus reduce the demand for international trade.

FDI: the regression coefficients of this variable show greater uncertainty in five models. In Model (2) and Model (3), this variable has a positive impact on the export of Zhejiang Province at the 5% significant level, while the regression results in Model (4) and Model (5) are significantly negative. This confirms the research theory about the relationship between FDI and international trade; that is, there are complementary and substitution relations between FDI and international trade, so there are uncertainties.

Land area and distance variables: the coefficients of these two variables are negative in the model. According to the gravity model, the farther the bilateral trade distance is, the higher the trade cost will be, thus leading to the reduction of trade volume. As can be seen from Model (3), the export volume of Zhejiang Province to the country declines by 0.79 units for every additional unit of distance, while a broader territory of the trading partner country means that the country has more abundant resources endowment, which prompts a reduction in the country’s import demand; therefore, distance is negatively correlated with export trade. According to Model (4), when the land area of the trading partner country increases by 1 unit, the export volume from Zhejiang Province to this country decreases by 0.41 units.

The human capital: the coefficient of this variable is positive in Model (5), and it is significant at the level of 10%. In Model (5), we can see that the export volume of Zhejiang Province enlarges by 0.027 units for every unit increase in human capital level. From the traditional economic theory, it can be deduced that a higher level of human capital will enhance the comparative advantage of the export region, thus promoting the development of export trade.

## 5. Conclusions, Recommendations, and Limitations

### 5.1. Conclusions

Firstly, the relationship between environmental regulation and the export of Zhejiang Province is U-shaped, which has a dynamic effect of first restraining and then promoting. There is a turning point, the value of which is −3.86. Before the inflection point of the U-shaped curve, environmental regulation has a negative correlation with export volume, meaning that the higher the level of environmental regulation, the great the harm to export. This is because in the initial stage, when the level of environmental regulation is low, enterprises control pollution emissions, improve the technological level, or purchase pollutant-discharge permits and pay environmental taxes to meet the standards, and these could increase the production costs of enterprises because of the implementation of environmental-regulation policy, resulting in a negative impact on export trade in the end. However, environmental regulation is positively related to the development of export trade after the inflection point of the U-shaped line. This is because reasonable environmental regulation can effectively promote enterprises to carry out a series of innovative activities, improve their competitiveness by utilizing the innovation compensation effect and first-mover advantage, and realize the coordinated development of environment and trade, which is supported by the famous “Porter hypothesis”. In addition, strict environmental regulation can benefit the trade structure through upgrading the industrial structure and regional transfer of polluting industries. Therefore, environmental regulation will offset the negative effects caused by the increase in trade costs in the earlier period by promoting trade innovation and optimizing the trade structure, thus promoting export trade in the long term.

Secondly, the GDP of Zhejiang Province and its trading partner countries and the level of human capital in Zhejiang Province have a positive impact on the development of its export. That is, the higher the GDP level of the two regions, the higher the level of human capital of Zhejiang Province, and the more conducive to the development of the export in Zhejiang Province. Thirdly, the distance between Zhejiang Province and its trading partner countries and the territorial area of the trading partner countries have a negative impact on the export of Zhejiang Province. In other words, the greater the distance between the two regions, the larger the size of the trading partner country, and the more unfavorable it is for exports. Fourthly, the impact of the population size of FDI in Zhejiang Province and its trading partner countries on export is uncertain. On the one hand, a larger population size mean that the region has a higher level of human capital or a larger market capacity, which stimulates the development of export. On the other hand, a larger population size is also more likely to promote domestic trade, thereby reducing the demand for international trade. At the same time, substitutional and complementary relationships exist between international trade and FDI. Therefore, the correlation between FDI, population size, and export still needs more research.

### 5.2. Policy Suggestions

#### 5.2.1. Coordinate Short-Term Goals with Long-Term Goals

As seen in the results of empirical analysis, the relationship between environmental regulation and export trade is U-shaped. Although it restrains the development of export trade in the short term, it will be beneficial to promote the development of export trade in the long term. Therefore, when implementing an environmental-regulation policy, local governments should formulate different principles in different stages according to their actual conditions. To achieve goals, we should coordinate the relationship between short-term and long-term goals. In the early stage of policy implementation, we should regard reducing environmental pollution as the main objective, strictly implement regulatory policies, and not relax environmental regulations because of the slowdown of export growth. When the policy is implemented to a certain stage, that is, when the regulatory level reaches the inflection point of the U-shaped curve, we should aim to improve the level of export as the main objective. The policy of border regulation should be adjusted around the growth of export to promote the coordinated development of the environment and trade.

#### 5.2.2. Improving the Policy System of Environmental Regulation

Currently, the intensity of environmental regulation in Zhejiang Province has not exceeded the inflection point of the U-shaped curve, which means that the improvement in environmental regulation intensity is not beneficial to the development of export trade. Thus, the government should strengthen the implementation of environmental-regulation policies, improve scientific methods of environmental management and the regulatory evaluation system, and support implementation by professional institutions. Before implementing each regulation, the government should ensure that investment is proportional to efficiency. For example, the government should execute related regulations according to the practice of existing regulations and effectively deal with the problem of excessive cost but low efficiency of regulation. In addition, local governments need to strengthen their autonomy in environmental management. Specifically, provincial governments should play a leading role in the environmental management system on the premise of maintaining a high degree of consistency with the central government in environmental regulation. This will not only help solve the problems whereby many single environmental policies in China fail to reflect the needs of environmental governance in different regions but also ensure that the provincial government, according to its own situation, clarifies the responsibilities and objectives of all levels and maximizes social and economic benefits while achieving the goal of environmental regulation.

#### 5.2.3. Diversification of Environmental Regulation Means

The environmental-regulation methods of the Zhejiang government are still dominated by the traditional command-and-control type, and this kind of governance approach often creates a problem of high investment and low efficiency, which aggravates the financial burden of the government. According to our conclusions, Zhejiang Province is still on the left side of the U-shaped curve. At this time, we should vigorously develop market-motivated environmental regulation, voluntary environmental regulation, and other similar mechanisms. Through market mechanisms such as sewage-discharge fees and emission permit trading, the externality effect of environmental regulation can be brought into play, and special technical subsidies for industrial industries should be strengthened to stimulate enterprise innovation, promote the production of green products and the development of new clean technologies, and exploit technological innovation to improve the comparative advantages of products, contributing to optimizing product structures. At the same time, we should actively encourage enterprises to formulate green strategies to gain initiative and first-mover advantage in the fierce competition in the future, which is favorable to optimizing trade partners, thus promoting the sustainable development of export.

#### 5.2.4. Improving the Level of Human Capital Investment

The role of human capital in promoting the development of export should not be neglected. Considering this, the government should increase its investment in human capital, strive to improve the stock and quality of human capital, breach the bottleneck of human capital restricting technological innovation, and reduce the negative impact of environmental regulation on export trade development by improving the innovation ability and performance of enterprises. Relevant government institutions should further intensify investment in high-end technical personnel while building a high-quality application-oriented, compound, and innovative personnel training system to provide strong talent and scientific and technological guarantees to promote industrial transformation, upgrading, and export trade development.

### 5.3. Limitations and Future Directions

Based on the panel data of Zhejiang Province and 18 major trading countries (regions) from 2004 to 2016, this paper empirically analyzes the impact of environmental regulation on export trade, and the result shows a U-shaped relationship. Before the U-shaped inflection point, since the intensity of environmental regulation is negatively correlated with the export trade volume, it is necessary to further study how to adjust the environmental regulation policy to reduce the negative effect on export trade. In the future, it is necessary to expand the research on the evaluation of the heterogeneity effect of environmental-regulation policies. At the same time, evaluations of the cost effect, innovation effect, and structural effect of environmental regulation are also worthy of further research.

## Figures and Tables

**Table 1:** Stationarity test result.

Variable	LLC	AD-Fisher
lnEX	−9.327 (0.0000) ***	4.141 (0.0000) ***
lnGDPZ	−10.645 (0.0000) ***	9.162 (0.0000) ***
lnGDP	−11.326 (0.0000) ***	11.044 (0.0000) ***
lnPOPZ	−14.882 (0.0000) ***	13.832 (0.0000) ***
lnPOP	−12.771 (0.0000) ***	20.905 (0.0000) ***
lndistance	−8.390 (0.0000) **	14.721 (0.0000) **
lnland	−18.322 (0.0000) ***	8.102 (0.0000) ***
lnRL	−17.665 (0.0010) **	18.971 (0.0000) **
lnFDI	−21.287 (0.0000) **	7.556 (0.0000) **
lnER	−19.868 (0.0024) ***	24.589 (0.0000) ***
(lnER)^2^	−16.519 (0.0000) ***	19.063 (0.0000) ***

Notes: **, and *** indicate significance at 5%, and 10%, respectively.

**Table 2:** Descriptive statistics.

Variable	Definition	Mean	Standard Deviation	Minimum	Maximum
lnEX	Export volume (logged)	3.97	0.84	2.08	6.19
lnGDPZ	GDP (logged)	7.92	0.89	5.81	8.87
lnGDP	GDP of trading partner countries (logged)	9.80	1.11	7.43	12.13
lnPOPZ	Population (logged)	8.45	0.30	7.45	8.63
lnPOP	Population in trading partner countries (logged)	8.51	0.95	6.52	10.38
lndistance	Distance (logged)	8.34	1.02	6.70	9.30
lnland	Territorial area (logged)	12.81	2.57	7.00	16.12
lnRL	Human capital (logged)	−5.12	0.38	−5.68	−4.47
lnFDI	FDI (logged)	7.79	3.80	−0.40	12.13
lnER	Environmental Regulation Level (logged)	−4.31	0.50	−5.43	−3.20
(lnER)^2^	Quadratic term of logarithm of environmental regulation level	18.79	4.31	10.23	29.49

**Table 3:** Results.

	(1)	(2)	(3)	(4)	(5)
L.EX	0.783 *** (0.024)	0.911 *** (0.017)	0.843 *** (0.008)	0.872 *** (0.011)	0.699 *** (0.005)
lnGDPZ	0.32 *** (3.41)	0.18 ** (2.31)	0.43 *** (5.71)	0.17 ** (2.01)	0.26 *** (3.66)
lnGDP	0.68 *** (3.71)	0.73 ** (2.07)	0.55 *** (2.58)	0.43 *** (3.31)	0.89 *** (3.67)
lnPOPZ	0.19 * (1.06)	0.27 *** (2.97)	0.11 ** (0.9)	−0.32 ** (−2.73)	−0.53 ** (−2.17)
lnPOP	0.13 ** (2.15)	0.09 *** (3.22)	0.18 ** (2.07)	0.24 ** (1.89)	0.41 ** (2.01)
lnER	0.24 ** (2.58)	0.35 ** (1.37)	0.25 ** (2.96)	0.16 ** (1.89)	0.18 ** (2.78)
(lnER)2	0.042 *** (2.18)	0.063 ** (2.27)	0.059 *** (3.13)	0.062 ** (2.91)	0.031 ** (2.21)
lnFDI		0.14 ** (0.87)	0.08 ** (1.83)	−0.09 ** (−1.01)	−0.19 ** (−1.38)
lndistance			−0.79 ** (−1.71)	−1.14 ** (−3.11)	−0.27 ** (−2.13)
lnland				−0.41 ** (−1.51)	−0.38 ** (−1.33)
lnRL					0.027 * (1.01)
constant	−3.89 ** (1.98)	−2.97 *** (1.79)	−2.03 ** (1.43)	5.58 ** (3.11)	1.96 *** (2.07)
AR (1)	−1.582 [0.020]	−1.411 [0.016]	−1.195 [0.008]	−1.897 [0.019]	−1.738 [0.013]
AR (2)	−0.373 [0.319]	−0.679 [0.412]	−0.521 [0.430]	−0.133 [0.293]	−0.248 [0.334]
Sargan Test	59.833 [0.513]	61.371 [0.709]	59.382 [0.383]	68.903 [0.258]	57.581 [0.681]

Notes: *** *p* < 0.01, ** *p* < 0.05, * *p* < 0.1; Z statistic values in parentheses; *p* values in brackets.
